# Alkylation of the Tumor Suppressor PTEN Activates Akt and β-Catenin Signaling: A Mechanism Linking Inflammation and Oxidative Stress with Cancer

**DOI:** 10.1371/journal.pone.0013545

**Published:** 2010-10-21

**Authors:** Tracy M. Covey, Kornelia Edes, Gary S. Coombs, David M. Virshup, Frank A. Fitzpatrick

**Affiliations:** Department of Medicinal Chemistry, University of Utah Health Sciences Center, Salt Lake City, Utah, United States of America; University of Illinois at Chicago, United States of America

## Abstract

PTEN, a phosphoinositide-3-phosphatase, serves dual roles as a tumor suppressor and regulator of cellular anabolic/catabolic metabolism. Adaptation of a redox-sensitive cysteinyl thiol in PTEN for signal transduction by hydrogen peroxide may have superimposed a vulnerability to other mediators of oxidative stress and inflammation, especially reactive carbonyl species, which are commonly occurring by-products of arachidonic acid peroxidation. Using MCF7 and HEK-293 cells, we report that several reactive aldehydes and ketones, e.g. electrophilic α,β-enals (acrolein, 4-hydroxy-2-nonenal) and α,β-enones (prostaglandin A_2_, Δ12-prostaglandin J_2_ and 15-deoxy-Δ-12,14-prostaglandin J_2_) covalently modify and inactivate cellular PTEN, with ensuing activation of PKB/Akt kinase; phosphorylation of Akt substrates; increased cell proliferation; and increased nuclear β-catenin signaling. Alkylation of PTEN by α,β-enals/enones and interference with its restraint of cellular PKB/Akt signaling may accentuate hyperplastic and neoplastic disorders associated with chronic inflammation, oxidative stress, or aging.

## Introduction

Inflammation and cancer are intricately linked [1], [2]. ‘Smoldering’ inflammation [3], also called para-inflammation [4], occurs in many types of pre-malignant and malignant tumors, e.g. colorectal adenoma and adenocarcinoma where the content of inflammatory leukocytes and the inflammatory enzyme cyclooxygenase-2 (COX-2) influence progression, prognosis and survival [5], [6]. Non-steroidal anti-inflammatory drugs (NSAIDs) that inhibit COX-2 can prevent certain, but not all, cancers [7]; and some NSAIDs, such as sulindac sulfone, act independently of COX and prostaglandin E_2_ (PGE_2_) inhibition [8]. Other NSAIDs, e.g. celecoxib, can paradoxically enhance tumor progression in APC^Min/+^ mice, which model intestinal tumorigenesis [9]. While COX-2 and its metabolite PGE_2_ are undoubtedly important, para-inflammation may enhance tumorigenesis by mechanisms that are incompletely understood. Innate immune mechanisms are prime candidates for investigation.

Usually, innate immune inflammation consists of a “wounding” phase to annihilate pathogens, and a “healing” phase to repair and regenerate damaged host tissue [10]. The transition between phases depends on gradual exhaustion of inflammatory mediators and conversion of certain pro-inflammatory mediators, e.g. PGD_2_, into anti-inflammatory metabolites, Δ12-PGJ_2_ [11], [12], [13]. Elements of the inflamed site itself, e.g. reactive oxygen species (ROS), albumin, fibroblasts and neutrophils, orchestrate this conversion [14], [15], [16], [17]. For example, reactive oxygen species (ROS) cause non-enzymatic peroxidation of essential fatty acids, like arachidonic acid (AA) [18]. AA hydroperoxides transform readily into reactive products containing an α,β–unsaturated carbonyl [19], [20] that include acrolein (2-propenal) [21], [22], 4-hydroxy-2-nonenal (4-HNE) [23], and cyclopentenone prostaglandins (cyPGs), PGA_2_ and Δ12-PGJ_2_ [24]. Covalent modification of NFκB and IKKα/β proteins by these α, ß–unsaturated carbonyl metabolites (i.e. protein alkylation) seems to be a “switch” to terminate inflammation [25]. Following this precedent, we hypothesized that alkylation may also act as a “switch” to initiate repair and regeneration of tissue damaged by inflammation.

PTEN (phosphatase tensin homolog on chromosome 10) is a phosphoinositide-3-phosphatase with two physiological roles: tumor suppressor and regulator of anabolic/catabolic cell signaling. The *PTEN* gene is frequently mutated or inactivated in advanced cancers [26]. Using MCF7 and HEK-293 cells, we report that reactive α, ß–unsaturated carbonyls (acrolein, 4-HNE, and Δ12-PGJ_2_) inactivate the PTEN protein – not the gene - by alkylation. Inactivation of PTEN by α, ß–unsaturated carbonyls leads to increased Akt signaling, enhanced nuclear β-catenin signaling, and augmented cellular proliferation. Redox signaling by PTEN may have evolved to enable cells (tissues) to stratify their response to oxidative stress. For example, transient inhibition of PTEN by reactive oxygen or carbonyl species, and the corresponding signaling through Akt/GSK3β/β-catenin/TCF4/Lef1 might benefit the host via increasing proliferation and regeneration of tissue damaged by acute inflammation or oxidative stress. Errant and persistent PTEN inactivation by the same molecular mechanism might favor tumor progression and provide an etiological link between ‘smouldering’ inflammation and certain cancers, especially colorectal cancer, where both the PTEN and the APC tumor suppressors restrict nuclear β-catenin signaling [27].

## Results

### The α, ß–unsaturated carbonyls acrolein, 4-HNE and Δ12-PGJ_2_ covalently modify cellular PTEN

**Figure 1:**
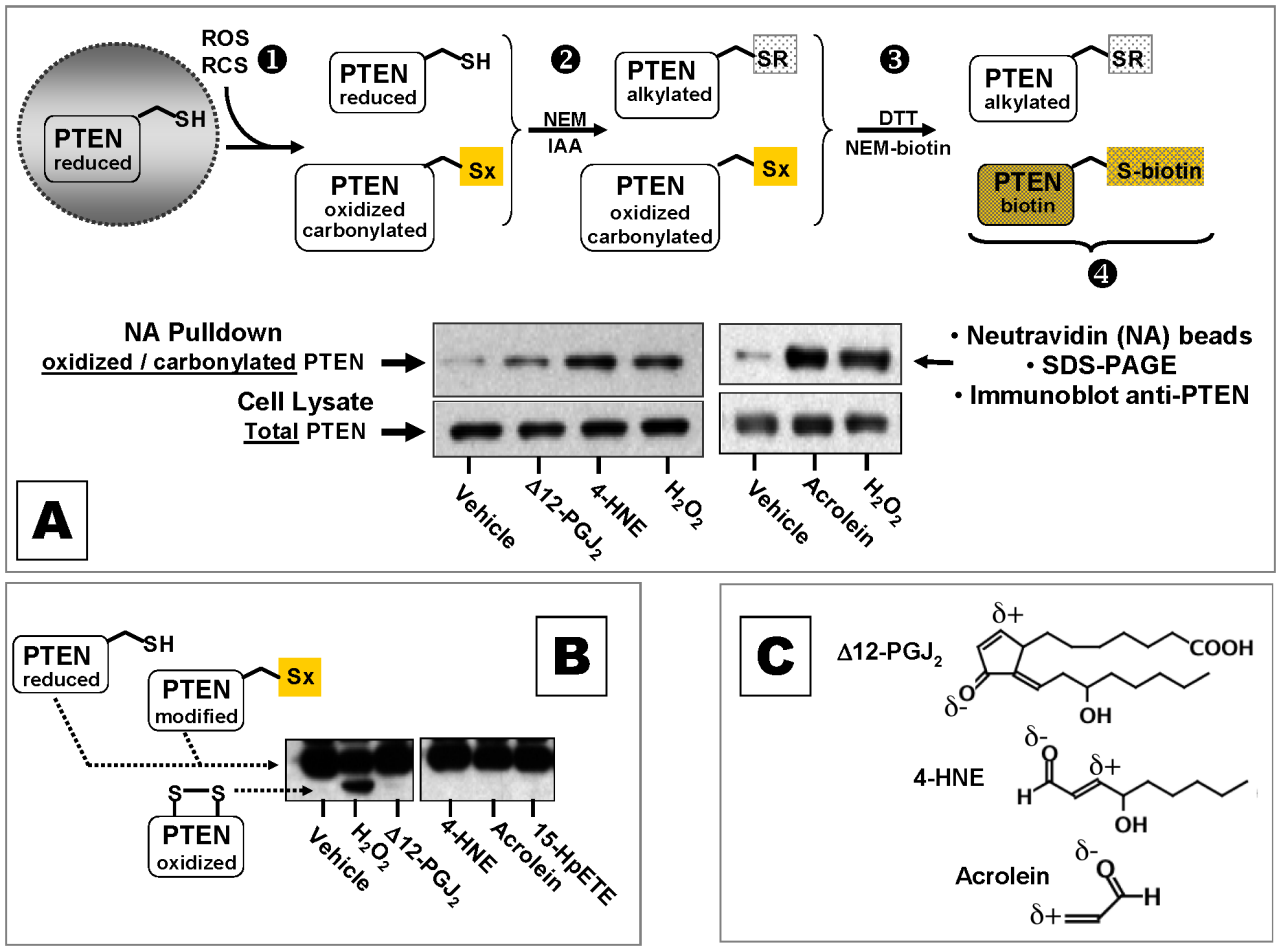
α, ß–unsaturated carbonyls covalently modify cellular PTEN. (**A**) Diagram of the procedure to identify PTEN with an oxidized or alkylated thiol in cells exposed to ROS or α, ß–unsaturated carbonyls. The anti-PTEN immunoblot shows oxidized or carbonylated PTEN (NA Pulldown) relative to total PTEN (Cell Lysate) isolated from MCF-7 cells treated 30 min with vehicle (DMSO), 10 µM Δ12-PGJ_2_ or 4-HNE versus 10 min with 100 µM H_2_O_2_; an immunoblot from a separate experiment shows oxidized, carbonylated and total PTEN in cells treated for 30 min with vehicle or 20 µM acrolein versus 10 min with 100 µM H_2_O_2_. (**B**) Anti-PTEN immunoblot of MCF-7 cell lysates fractionated by SDS-PAGE under non-reducing conditions. PTEN oxidized to a Cys^124^-Cys^71^ disulfide appears as a faster migrating species (PTEN oxidized disulfide) only in cells treated with H_2_O_2_. This species was undetectable in MCF-7cells treated 30 min with DMSO vehicle or 20 µM each Δ12-PGJ_2_, 4-HNE, acrolein or 15-HpETE. (**C**) Chemical structures of typical α, ß–unsaturated carbonyls. Acrolein and 4-HNE are α, β enals; Δ12PGJ_2_ is an α,β enone. Electrophilic β carbons are denoted with δ^+^. Blots are representative of results obtained in at least three independent experiments.

We exposed MCF-7 cells to representative α, ß–unsaturated carbonyl (**Figure 1C**) or H_2_O_2_, then selectively tagged any proteins that had oxidized or carbonylated thiols using NEM-biotin **(** **Figure 1A** **)**. We then sequestered proteins with a biotin epitope onto NeutrAvidin (NA) beads and identified carbonylated PTEN by SDS-PAGE and immunoblotting. MCF-7 cells treated with 10 µM Δ12-PGJ_2_, 4-HNE, or acrolein contained carbonylated PTEN in amounts comparable to cells treated with 100 µM H_2_O_2_ (**Figure 1A** **,** NA pulldown**)**. This method does not distinguish between oxidized and carbonylated thiols on PTEN. However, electrophoresis under non-reducing conditions, followed by western blotting, showed that PTEN migrated as a discrete isoform due to an oxidized disulfide [28], [29], which occurred only in cells treated with 100 µM H_2_O_2_, but not in cells treated with α, ß–unsaturated carbonyl (Δ12-PGJ_2_, 4-HNE, acrolein) or 15-HpETE, a lipid hydroperoxide (**Figure 1B**).

### Cyclopenteneone PG-biotin analogs are model α, β–enones that alkylate PTEN

**Figure 2 pone-0013545-g002:**
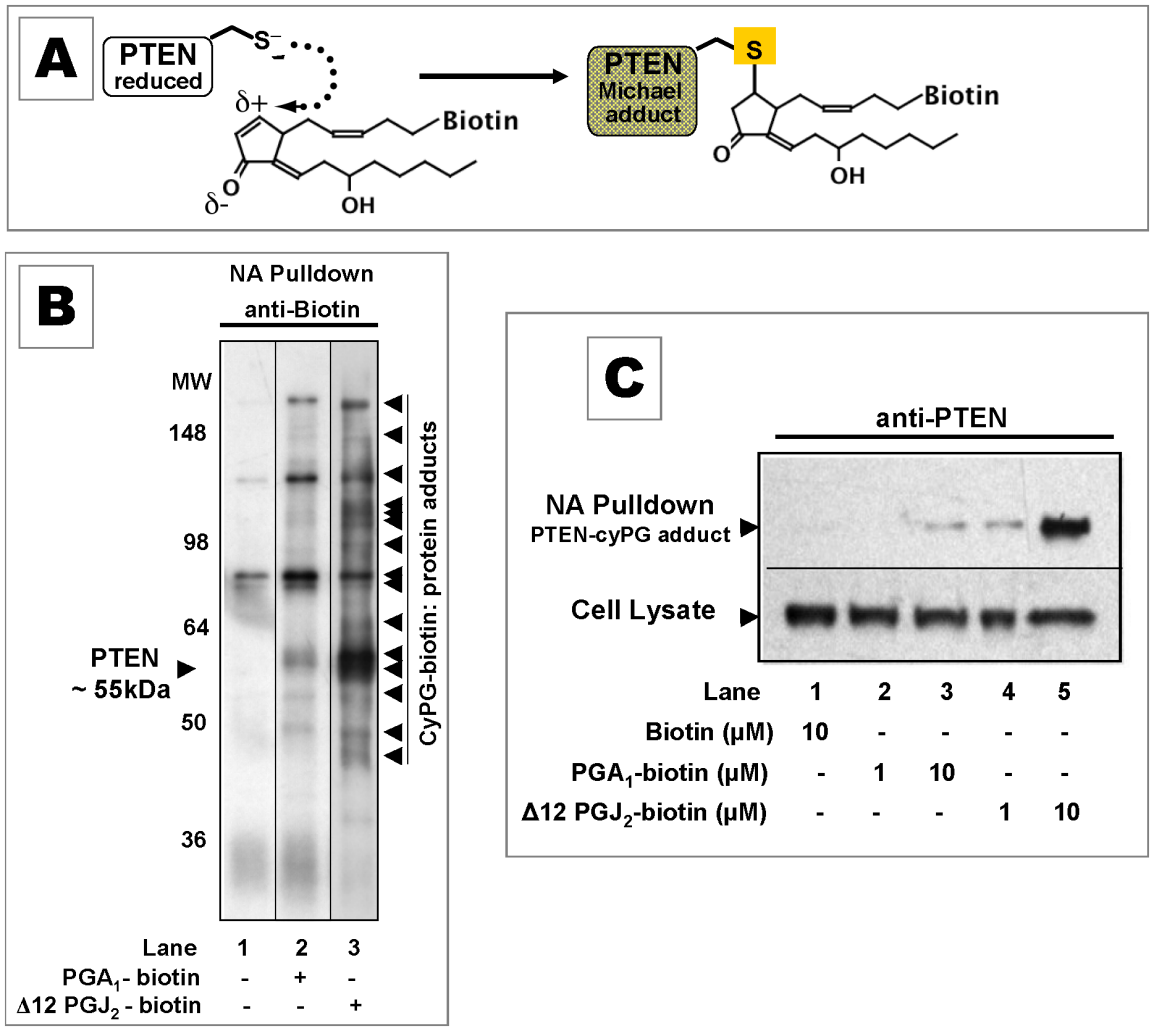
CyPG biotin analogs: model α, ß–unsaturated carbonyls alkylate cellular PTEN. (**A**) Michael addition reaction between PTEN and a Δ12-PGJ_2_-biotin analog. Following treatment of cells with cyPG-biotin, proteins with a *de novo* biotin epitope were sequestered onto neutravidin beads (NA Pulldown), then fractionated by SDS-PAGE for immunoblotting. (**B**) Anti-biotin immunoblot of proteins from lysates of MCF-7 cells treated with DMSO (lane 1), 10 µM PGA_1_-biotin (lane 2), and 10 µM Δ12PGJ_2_-biotin (lane 3). Δ12-PGJ_2_ biotin formed a covalent adduct with proteins more readily than PGA_1_-biotin (arrowheads). (**C**) Anti-PTEN immunoblot of cellular PTEN that formed a covalent adduct with cyPG-biotin (NA Pulldown) relative to total PTEN (Cell Lysate) from MCF-7 cells treated with DMSO (lane 1), 1 or 10 µM PGA_1_-biotin (lanes 2, 3), and 1 or 10 µM PGJ_2_-biotin (lanes 4, 5). Blots are representative of results obtained in at least three independent experiments.

The cysteinyl thiolate in the PTEN active site (–HC(X_5_)RT–) is prone to oxidation because it is a strong nucleophile, pKa ∼5. This trait should also facilitate alkylation of PTEN by α, ß–unsaturated carbonyls. We used CyPG-biotin analogs, which have an electrophilic β-carbon capable of nucleophilic addition (Michael reaction), as chemical models to test this hypothesis [30]. Alkylation of any cellular proteins by these analogs would introduce a biotin epitope *de novo* (**Figure 2A**). PGA_1_-biotin and Δ12 PGJ_2_-biotin were both taken up into MCF-7 cells and formed covalent adducts with ∼20 proteins (**Figure 2B**). Δ12 PGJ_2_-biotin (a bi-functional dienone) was more reactive than PGA_1_-biotin (mono-functional enone), agreeing with others who reported ∼20-30 protein targets modified by cyPG-biotin in 3T3 cells or mitochondria [31], [32]. Sequestration of *de novo* biotinylated cellular proteins on NA beads, followed by immunoblot with anti-PTEN antibodies, showed that PTEN formed a covalent adduct ∼10-fold more readily with Δ12-PGJ_2_-biotin than with PGA_1_biotin (**Figure 2C** **,** lane 4 vs lane 3).

### The α, ß–enone, Δ12-PGJ_2_, interferes with PTEN suppression of Akt kinase

**Figure 3:**
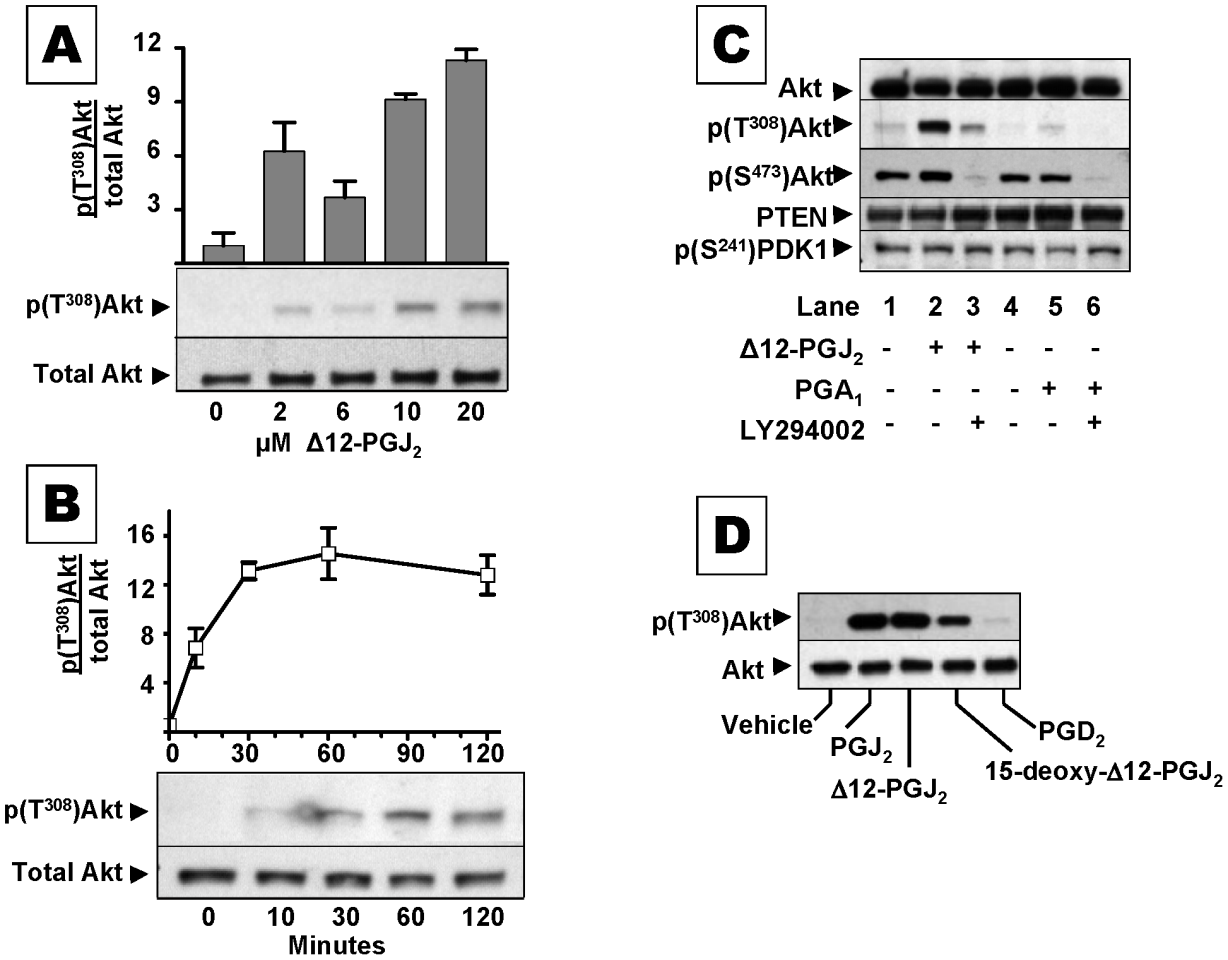
The α, ß–unsaturated enone Δ12PGJ_2_ interferes with PTEN suppression of Akt activation. (**A**) Immunoblots of phospho-(T^308^)Akt relative to total Akt in lysates from MCF-7 cells treated 30 min with 0–20 µM Δ12-PGJ_2_. The bar graph shows the increase in phospho-(T^308^)Akt/total Akt (mean ± s.e.m) from n≥3 separate experiments. (**B**) Immunoblot of phospho-(T^308^)Akt relative to total Akt in lysates from MCF-7 cells treated with 20 µM Δ12-PGJ_2_ for 0–120 min. The bar graph shows the increase in phospho-(T^308^)Akt/total Akt (mean ± s.e.m) from n = 4 separate experiments. (**C**) Immunoblots of total Akt, phospho-(T^308^)Akt, PTEN, phospho-(S^241^)PDK1 from lysates of MCF-7 cells treated 30 min with 20 µM Δ12-PGJ_2_ (lane 2, 3) and 20 µM PGA_1_ (lane 5, 6) in the presence of the PI3-K inhibitor 50 µM Ly294002 (lane 3, 6) or DMSO vehicle (lane 2, 5). (**D**) Immunoblot of phospho-(T^308^)Akt relative to total Akt in lysates from MCF-7 cells treated 4 hrs with vehicle, the cyclopentenones - PGJ_2_, Δ12-PGJ_2_, and 15-deoxy-Δ12-PGJ_2_, or their precursor PGD_2_. For (C) and (D), blots are representative of results obtained in three independent experiments.

Growth factors, insulin, and other stimuli prompt PI3-K to make PIP_3_, which recruits PKB/Akt kinase to the cell membrane where PDK1/2 phosphorylates Akt Thr^308^ and Akt Ser^473^ residues, respectively [33], [34], [35]. PTEN down-regulates PKB/Akt activation by metabolizing PIP_3_ to PIP_2_ [36]. α, ß–unsaturated carbonyls that alkylate cellular PTEN may interfere with its suppression of Akt kinase. A representative α, ß–enone, Δ12-PGJ_2_, caused a concentration and time dependent increase in phospho-(T^308^) Akt in MCF-7 cells. As little as ∼2 µM Δ12-PGJ_2_ caused a half-maximal response (**Figure 3A**). Increases in cellular phospho-(T^308^)Akt were detectable at 10 min, maximal at 30 min, and durable for >120 min (**Figure 3B**). Δ12-PGJ_2_ increased formation of phospho-(T^308^)Akt without altering formation of phospho-(S^241^)PDK1 (the kinase that phosphorylates T^308^ of Akt), and without altering PTEN protein expression (**Figure 3C**). These data suggest that Δ12-PGJ_2_ interfered with PTEN's capacity to restrain activation of Akt kinase. Consistent with this interpretation, co-treatment of cells with cyPGs plus 50 µM LY294002, lowered levels of phospho-(T^308^)Akt by inhibiting PI3-K, at the apex of the PI3-K/→PDK1/2→Akt kinase cascade (**Figure 3C** **,** lanes 3 vs 2, and 6 vs. 5). Furthermore, 1 µM of various PGA and PGJ isomers, and other α, ß–enones directly inhibited isolated PTEN enzyme [**Table 1**]. The electrophilic β carbon of cyPGs is essential for inhibition, since a structurally similar, but non-electrophilic cyPG, PGB_1_, was inactive.

**Table 1:** 

Test Compound	PTEN Activity
	**% Inhibition**
Vehicle	0
10 µM PGB_1_	5±2
1 µM PGA_1_	37±5
1 µM PGA_2_	40±3
1 µM PGJ_2_	48±5
1 µM Δ12-PGJ_2_	56±4
1 µM 15-deoxy-Δ12, Δ12-PGJ_2_	73±3
10 µM Acrolein	40±6
10 µM 4-hydroxy-2-nonenal	57±2

Mean ± sem, n = 3.

Structure-activity experiments showed that various J-series cyPGs, including PGJ_2_, Δ12 PGJ_2_ and 15-deoxy- Δ12, 14-PGJ_2_, increased phospho-(T^308^)Akt in MCF-7 cells, compared to vehicle or their precursor PGD_2_ (**Figure 3D**). PGA_1_ had a modest effect on cellular Akt phosphorylation (**Figure 3C** **, lane 5**), which corresponds with its weaker covalent modification of cellular PTEN (**Figure 2C** **, lane 3**).

**Figure 4:**
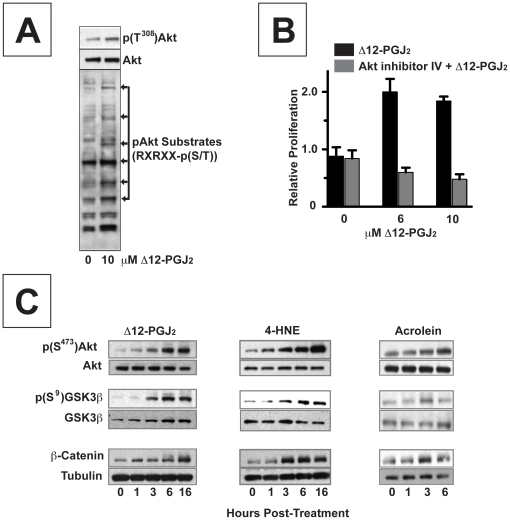
α, ß–unsaturated carbonyls interferes with Akt kinase and downstream signaling in MCF7 and HEK 293 cells. (**A**) Immunoblots of phospho-(T^308^)Akt, total Akt, and several phosphoproteins with (K/R)-x-(K/R)-xx-(S/T), a motif recognized and phosphorylated by active phospho-(T^308^)Akt, in lysates from MCF-7 cells treated 0 and 10 µM Δ12-PGJ_2_. (**B**) Relative proliferation of MCF-7 cells (mean ± s.e.m, n = 4) treated with 0, 1, and 10 µM Δ12-PGJ_2_ alone (▪), or in the presence of 10 µM of inhibitor IV (▪), an Akt kinase inhibitor. (**C**) Immunoblots of phospho-(S^473)^Akt, total Akt; phospho(S^9^)GSK3β, total GSK3β; and β-catenin and tubulin in lysates from HEK 293 cells after treatment for 0,1, 3, 6 and 16 hrs with 6 µM Δ12PGJ_2_, 6 µM 4-HNE or 20 µM acrolein. For (A) and (C), blots are representative of results obtained in three independent experiments.

Activated phospho-(T^308^/S^473^)Akt kinase can phosphorylate different protein substrates that regulate cell proliferation and fate [35]. Phosphorylation of Akt substrates with an RxRxx-phospho-S/T epitope, coincided with increased phospho-(T^308^)Akt in MCF-7 cells treated with 10 µM Δ12-PGJ_2_ (**Figure 4A**). Akt kinase activation also coincided with Akt-dependent proliferation in MCF-7 cells treated with 1–10 µM Δ12-PGJ_2_ (**Figure 4B** **)**. This finding is consistent with reported bi-phasic actions of cyPGs, whereby they increase proliferation of cultured cells at ∼1 µM [37], [38], while they decrease proliferation or cause apoptosis by modifying other protein targets at ∼50 µM [39].

### α, ß–unsaturated carbonyls cause a time-dependent accumulation of cellular phospho-(S^473^)Akt kinase (active) **→** phospho-(S^9^)GSK3β (inactive) → β-catenin and a rise in nuclear β-catenin signaling

**Figure 5:**
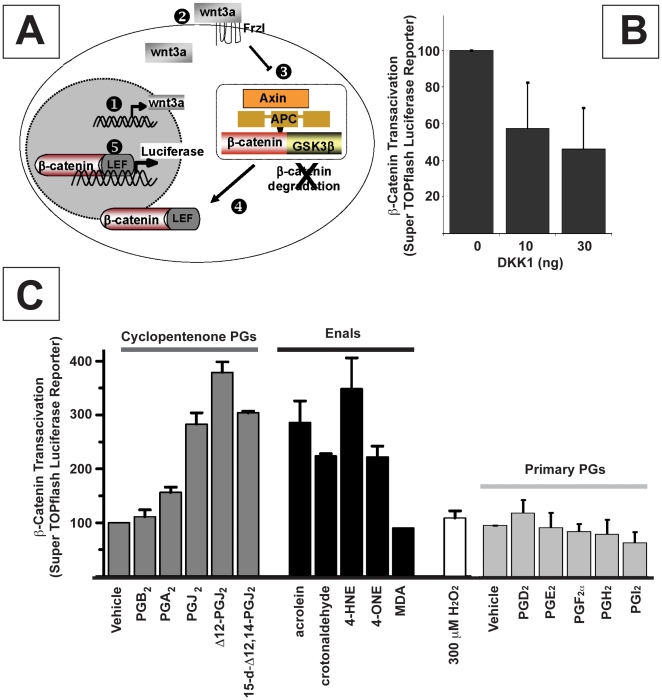
α, ß–unsaturated carbonyls enhance nuclear β-catenin signaling in STF3A cells: structure activity relationships. (**A**) STF3A cells harbor a stably integrated transgene which expresses WNT3A (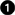). When secreted, WNT3A elicits autocrine/paracrine stimulation of Fzl receptors (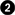) which inhibits APC-dependent turnover of β-catenin (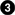). If β-catenin is not phosphorylated by GSK3β, it can accumulate as a β-catenin:LEF dimer (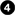) and bind to promoters on stably integrated β-catenin:luciferase reporter gene (SuperTop Flash) (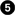). Luciferase activity in cell lysates is proportional to nuclear β-catenin signaling. (**B**) Nuclear β-catenin signaling (luciferase luminescence) in STF3A cells (2×10^4^ cells/well) grown for 24 hrs in medium containing 0, 10, or 30 ng/ml of DKK1 (mean + s.d., n = 3). DKK1, a wnt antagonist, inhibited β-catenin signaling (**C**) Nuclear β-catenin signaling in STF3A cells (2×10^4^ cells/well) grown for 24 hrs in medium containing 20 µM of cyPGs; α,β-enals or primary PGs. Several reactive electrophiles enhanced β-catenin signaling by ∼2–4 fold. Histogram represents the mean + s.e.m., n = 4.

GSK3β converts β-catenin to phospho-(S^33/37^/T^41^) β-catenin, which is rapidly eliminated by the 26S proteasome [40]. GSK3β acts in concert with the tumor suppressor APC. In cells with mutant APC, or when WNT ligands stimulate cells with wild type APC, GSK3β fails to phosphorylate β-catenin, which allows it to accumulate, associate with other nuclear transcription factors and express its target genes (e.g. *c-myc*, *cyclin D1*) [41]. PTEN can block β-catenin accumulation/signaling by favoring retention of active GSK3β and inactive PKB/Akt kinase in some [42], [43], but not all experimental systems [44], [45]. Accordingly, inactivated PTEN should augment β-catenin signaling by favoring retention of inactive phospho-(S^9^)GSK3β and active phospho-(S^473^) Akt kinase. We thus hypothesized that these electrophilic mediators may affect ß-Catenin signaling through this mechanism. To investigate this further, we used HEK 293 cells which have an intact ß-Catenin signaling pathway. We found that the different α, ß–unsaturated carbonyls that alkylated PTEN (6 µM Δ12 PGJ_2_, 6 µM 4-HNE and 20 µM acrolein) all caused a time-dependent rise in phospho-(S^473^)Akt (i.e. active Akt kinase), with a corresponding rise in phospho-(S^9^)GSK3β (i.e. inactive GSK3β) and β-catenin (**Figure 4C**). To determine if α, β–unsaturated carbonyls enhanced nuclear β-catenin signaling, we used HEK 293 cells engineered to stably express WNT3A and SuperTopFlash (STF) reporter gene [46]. These cells, called STF3A cells, thus secret WNT3A, an autocrine/paracrine stimulus for FZD receptors that slows APC-dependent degradation of β-catenin (**Figure 5A**). Nuclear β-catenin signaling in STF3A cells is proportional to their luciferase expression (activity), and they are responsive to DKK1, a WNT antagonist that inhibited β-catenin signaling in STF3A cells in a concentration-dependent manner (**Figure 5B**).

Several reative carbonyl metabolites, each with an electrophilic α,β enone or enal substituent, enhanced expression of the β-catenin:luciferase reporter gene in STF3A cells (**Figure 5C**). Luciferase reporter activity rose by ∼4-fold over baseline (p<0.01) in STF3A cells incubated with Δ12 PGJ_2_ or 4-HNE; by ∼3-fold (p<0.01) in cells with other PGJ analogs, acrolein or 4-ONE; and by ∼1.5-fold (p<0.05) in cells with PGA_2_. Consistent with our mechanistic hypothesis, neither PGB_2_ nor MDA had a detectable effect. PGB_2_ is a cyPG, but tautomerism prevents the charge de-localization required to create an electrophilic β carbon, which is required for protein alkylation. MDA (β-hydroxy-acrolein) penetrates cell membranes poorly because it is >99% ionized at physiological pH ∼7.4 used in our experiments. Neither PGE_2_ nor other primary PG metabolites of COX-1 or -2 had any effect on nuclear β-catenin signaling in STF3A cells. We draw attention to the fact that ectopic over-expression of EP receptors in HEK 293 cells was required to elicit any PGE_2_ mediated β-catenin signaling [47]. The weak response to PGE_2_ and other PG's in **Figure 5C** may reflect the constitutive levels of EP, FP, DP or IP receptors in STF3A cells or rapid metabolism of PGs, or both.

**Figure 6:**
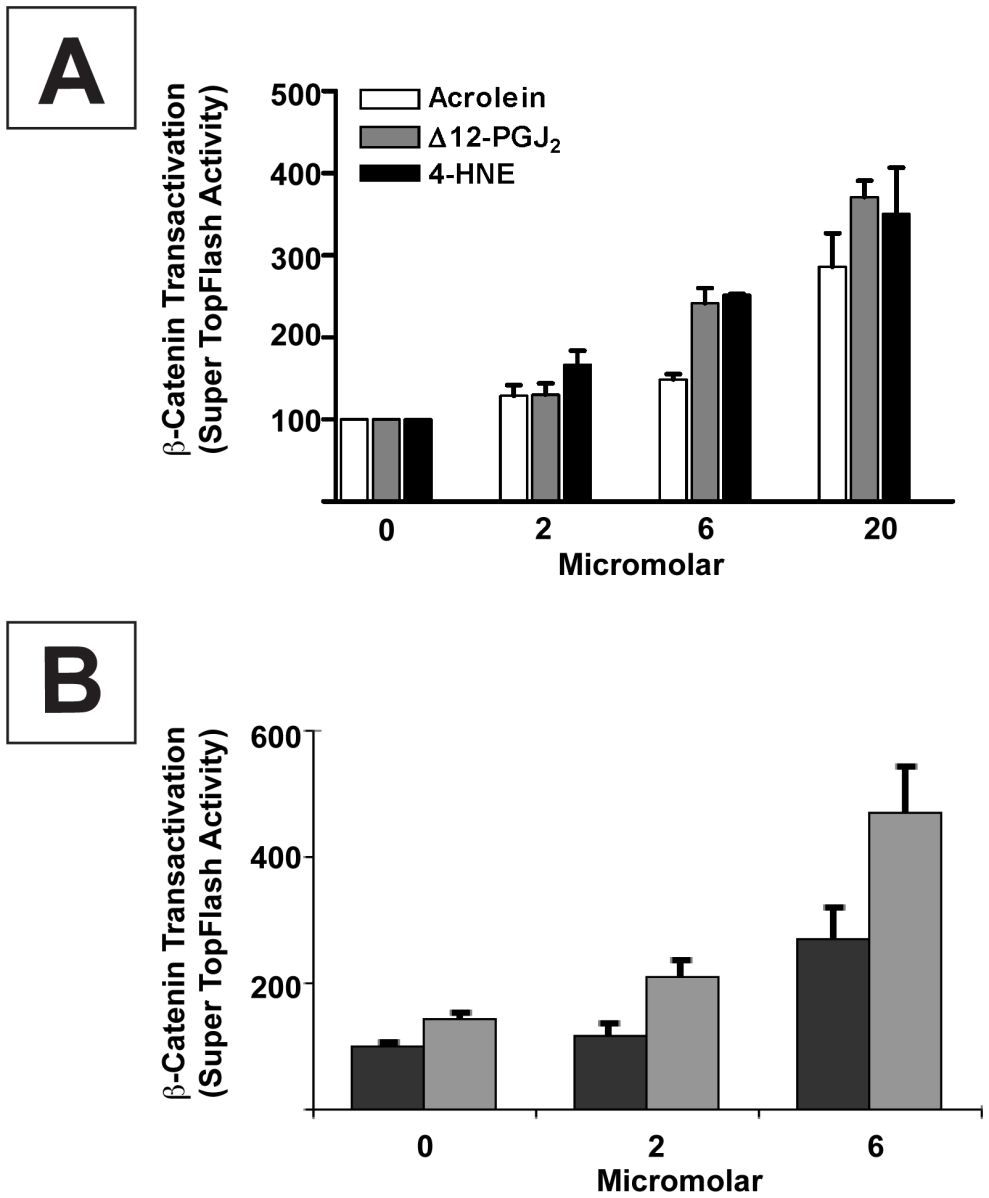
α, ß–unsaturated carbonyls enhance nuclear β-catenin signaling in STF3A cells:concentration-response relationships. (**A**) Nuclear β-catenin signaling (luciferase luminescence) in STF3A cells (2×10^4^ cells/well) grown for 24 hrs in medium containing 0, 2, 6 and 20 µM each of acrolein (□), Δ12 PGJ_2_ (▪) and 4-HNE (▪). (**B**) Nuclear β-catenin signaling in STF3A cells (2×10^4^ cells/well) grown for 24 hrs in medium containing 0, 2 or 6 µM of Δ12 PGJ_2_ alone (▪) or Δ12 PGJ_2_ plus 100 µM BSO. GSH levels fell by 79% and 88% at 6 and 24 hrs after treating cells with BSO; 92–95% of cells were still viable at 24 hrs. Bars represent the mean ± s.e.m from n≥3 separate experiments.

Enhanced β-catenin signaling in STF3A cells was concentration dependent between 2–20 µM for acrolein, 4-HNE and Δ12 PGJ_2_ (**Figure 6A**). Depletion of cellular GSH to ∼10% of baseline by treatment with 100 µM BSO potentiated β-catenin signaling, e.g. in STF3A cells treated with 2 and 6 µM Δ12 PGJ_2_ (**Figure 6B**). This is consistent with the role of reduced glutathione in the conjugation of reactive metabolites, and protection of redox sensitive proteins from alkylation [20].

## Discussion

The *PTEN* tumor suppressor gene is frequently mutated or inactivated in advanced cancers [26], [48]. PTEN is a phosphoinositide-3-phosphatase that metabolizes PIP_3_ to PIP_2_ [36], thereby counter-regulating PKB/Akt, a serine/threonine kinase proto-oncogene that controls anabolic growth and specification of cell fate [33], [34], [35]. PTEN, itself, is regulated post-translationally by phosphorylation [49], acetylation [50], and reversible oxidation of its catalytic cysteine^124^ residue [28], [29]. Oxidation of cellular PTEN can involve H_2_O_2_ derived from NADPH oxidase [51], superoxide dismutase [52], or enzymatic peroxidation of arachidonic acid (AA) by COX-1, COX-2 or 5-LOX [53]. All of these enzymes are commonly over-expressed and activated by inflammation or neoplastic transformation. PTEN oxidation, and any attendant pathophysiology, varies with the degree of cellular exposure to reactive oxygen species (ROS). In these studies, we demonstrate that the chemistry which facilitates oxidation of PTEN can also facilitate its alkylation by electrophilic α, ß-enals and α, β-enones [19], [20].

PTEN epitomizes the adaptation of redox-responsive thiols for cell signaling, as well as their potential vulnerability to by-products of oxidative stress and inflammation. PTEN is inactivated by two distinctive redox-mediated processes: 1) intra- or inter-molecular disulfide formation by ROS and 2) thiolate carbonylation (Michael addition) by electrophilic α, ß–unsaturated carbonyls (Figure 1–3). Hydrogen peroxide (H_2_O_2_), a prototypical ROS, inhibits cellular PTEN by directly oxidizing its catalytic Cys^124^ to a sulfenic acid intermediate, which then forms an inactive, intra-molecular Cys^124–71^ disulfide [28], [29]. Our data show that several representative, electrophilic carbonyl species (α,ß-enals and α,ß-enones), which can occur endogenously as byproducts of lipid peroxidation during inflammation or oxidative stress, alkylate and inactivate PTEN. Inactivation of PTEN by redox-mediated processes causes an increase in activity of the proto-oncogene Akt. Hyperactivation of Akt increases proliferation and survival of many different cancers.

Signaling by H_2_O_2_ spans a wide pathophysiological continuum [54] and a comparable role for reactive electrophiles seems plausible. Reactive carbonyl species such as acrolein, 4-HNE and Δ12PGJ_2_ represent a sub-set of electrophiles commonly produced during oxidative stress and inflammation. These findings might extrapolate to electrophilic agents lacking a carbonyl but containing other electron withdrawing groups, and we refer to them generally as “reactive electrophiles”. First, like H_2_O_2_, reactive electrophiles occur *in vivo* during inflammation and oxidative stress [16], [18], [19], [22]. Second, reactive electrophiles covalently modulate other proteins that regulate important signaling processes; i.e. LKB1/STK11 [55], NFκB [56], and IKKβ. Third, H_2_O_2_ and reactive electrophiles both originate from a combination of spontaneous and enzymatic processes, which often coincide in inflamed tissues [57]. H_2_O_2_ derives from superoxide anion, O_2_^−^, the primary metabolite of NADPH oxidases. Spontaneous and enzymatic dismutation converts O_2_^−^ into H_2_O_2_. Likewise, spontaneous and enzymatic lipid peroxidation generates acrolein and 4-HNE [18], [57]. cyPGs originate from the lipid endoperoxide PGH_2_, the primary metabolite of COX-1 and -2. Enzymatic and spontaneous scission of endoperoxide bonds converts PGH_2_ into PGE_2_ and PGD_2;_ albumin/serum then causes their dehydration into PGA_2_, PGJ_2_, and their isomers [14], [16], [24].

**Figure 7:**
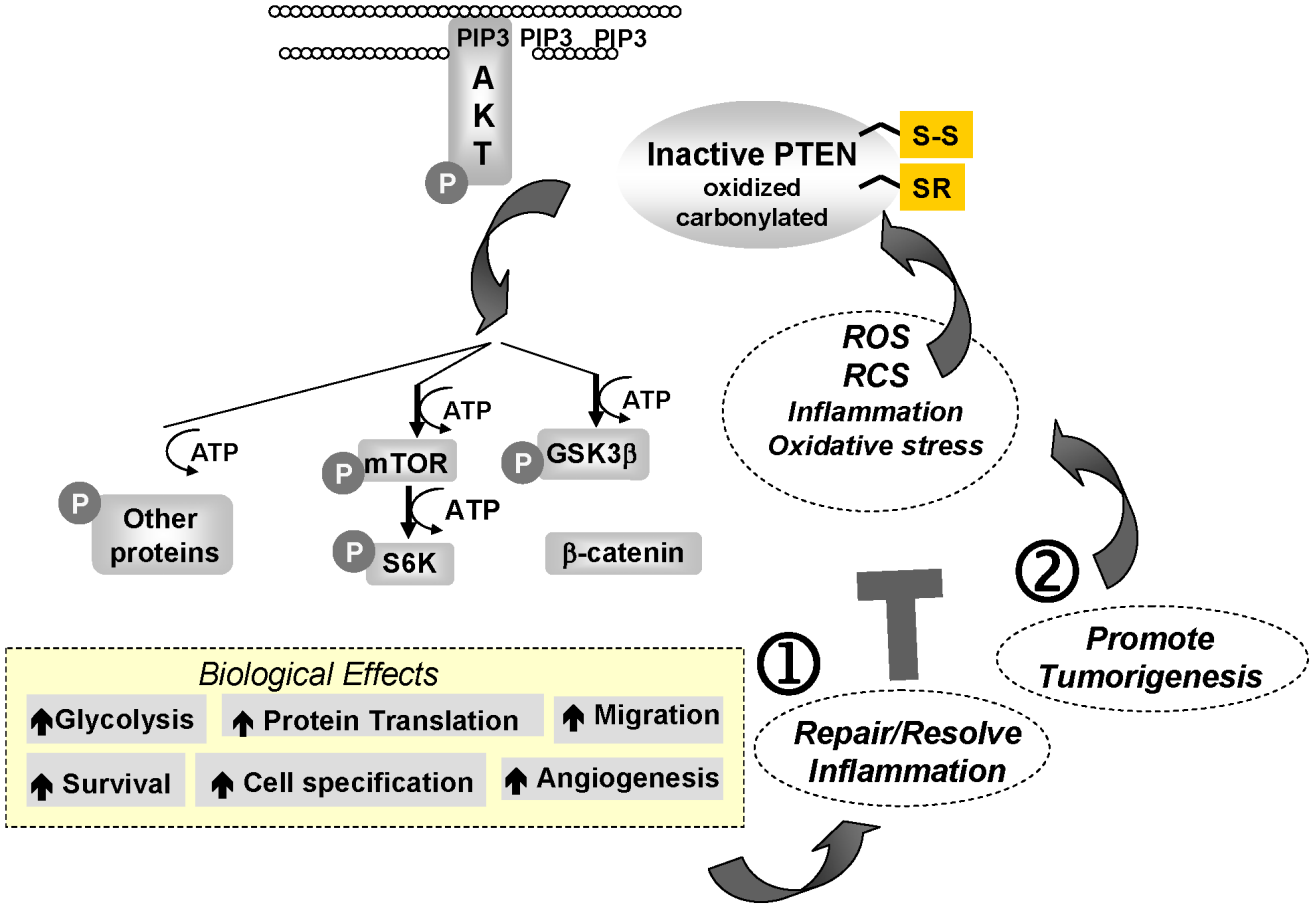
Model Depicting Hypothetical Roles of PTEN Alkylation in Inflammation and Cancer.

While speculative, it appears that ROS and reactive electrophiles (H_2_O_2_, acrolein, 4-HNE, Δ12 PGJ_2_) may have both evolved to play disparate roles in innate immunity: 1) annihilating pathogens and 2) resolving inflammation. Analogous to inactivation of NFκB and IKKαβ, temporary inactivation of the PTEN tumor suppressor protein by its alkylation, and attendant activation of PKB/Akt kinase proto-oncogenes, might help normalize morphology and histology at acutely inflamed tissues by releasing their restriction on cell proliferation, anabolic growth and fate specification [34], [35]. In ordinary situations repair and resolution should help terminate innate immune inflammation (**Figure 7** **,
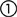
**). However, this mechanism might also confer inescapable risks if PTEN were inactivated errantly or persistently. Furthermore, reactive electrophiles also inactivate other notable tumor suppressors, including p53 [30] and LKB1/STK11 [55]. This combined and sustained inactivation of tumor suppressors could contribute significantly to inflammation-associated tumorigenesis and subsequently prolong the cycle of tumor-associated para-inflammation (**Figure 7** **,
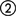
**). Overall, our data and model align with the observation that tumors are wounds that fail to heal [58]. In this situation, tumor progression may derive partly from mal-adaptation of a molecular mechanism that evolved to terminate and resolve innate immune inflammation.

Inflammation is a critical component of tumor progression. Many cancers arise from sites of infection, chronic irritation and inflammation. The tumor microenvironment, which is comprised largely of inflammatory cells, plays a major role in the neoplastic process, fostering proliferation, survival, and migration. We show herein that reactive carbonyl species that are commonly produced during inflammation covalently modify and inactivate PTEN tumor suppressor. Importantly, the mechanism we describe might also extrapolate to: 1) other electrophilic species generated by inflammation, oxidative or xenobiotic stress (i.e. other α, ß unsaturated aldehydes and ketones; allylic or vinyl epoxides; quinones, chlorhydrins, chloramines, vinyl sulfones; and 2) other members of the PTP superfamily that are redox sensitive. These studies extend our understanding of the mechanisms by which inflammation contributes to the initiation and progression of cancer.

## Materials and Methods

### Materials

We used minimum essential medium (MEM), supplements, bovine insulin, gentamicin, human embryonic kidney HEK-293 cells, and HEK-293 cells containing the Epstein Barr virus nuclear antigen 1 gene (HEK-EBNA1) (Invitrogen; Carlsbad, CA); MCF-7 cells (HTB-22, American Type Culture Collection; Manassas, VA); PGs, cyPG-biotin analogs, and WST proliferation assay kits (#10008883) (Cayman Chemical; Ann Arbor, MI); Complete™ protease inhibitor mixture (Roche Molecular Biochemicals; Indianapolis, IN); lysis buffer 0.6% Igepal CA-630 in PBS (Promega; Madison, WI); NeutrAvidin conjugated beads, NEM-biotin and goat anti-biotin polyclonal antibodies (#31852) (Pierce Chemical; Rockford, IL); a PI3-K inhibitor, LY294002 (# 9901), polyclonal antibodies against PTEN (#9552), Akt (#9271), phospho-(Thr^308^)Akt (#9375), phospho-(S^473^)Akt (#9271), phospho-(S^9^)GSK3β (#9336), GSK-3β (# 9332), phospho-(Ser^33/37^/Thr^41^) β catenin (#9561S) K/R-x-K/R-x-x-S/T(PO_4_) epitopes (#9614) and phospho-(S^241^)PDK1 (#3061) (Cell Signaling Technologies; Danvers, MA); β-catenin (# C19220)(BD Transduction Laboratories; Franklin Lakes, NJ); HRP (horseradish peroxidase) conjugated secondary antibodies (Santa Cruz Biotechnology; Santa Cruz, CA); polyvinylidene difluoride (PVDF) membranes and Western Lightning™ chemiluminescence reagents (Perkin-Elmer; Waltham, MA); PTEN enzyme assay kits (# 17–351)(Upstate Biotechnology; Lake Placid, NY); Akt inhibitor IV (#124011) (Calbiochem; San Diego, CA); acrolein (#01680), crotonaldehyde (# 262668), L-Buthionine-sulfoximine (BSO) (B2515), H_2_O_2_ 30% solution (H-1009), malondialdehyde (Fluka Cat.# 63287) (Sigma-Aldrich; St Louis, MO); DKK-1 (#1096-DK-010) (R & D Systems; Minneapolis, MN) and Luciferin (Cat.# 10101-2) (Biotium Inc., Hayward, CA).

### Cell culture

MCF-7 breast cancer cells (ATCC) were grown in MEM with 10% v/v FBS, 2 mM L-glutamine, 1.5 g/l NaHCO_3_, 0.1 mM non-essential amino acids, 1 mM sodium pyruvate, 0.01 mg/ml bovine insulin, and 0.01 mg/ml gentamicin. HEK-293 cells (ATCC) were grown in MEM with 10% fetal calf serum, 100 units of penicillin/streptomycin, 2 mM L-glutamine, and 1 mM pyruvate.

### Identification of modified PTEN by tagging with biotin-conjugated maleimide

MCF-7 cells (MEM 1% v/v FBS) were treated for 30 min with vehicle, or reactive electrophiles (10 µM Δ12-PGJ_2_, 4-HNE, acrolein), or 100 µM H_2_O_2_. Media was removed; cells were frozen at −80°C for 15 min; transferred to vacuum and incubated 1 h, 25°C with 1 ml of O_2_-free extraction buffer (50 mM NaHPO_4_, pH 7.0, 1 mM EDTA, 10 mM NEM [N-ethyl maleimide], 10 mM IAA [iodoacetic acid], 1% Triton X-100, 5 mM NaF, 50 µg/ml leupeptin and 50 µg/ml aprotinin). This treatment selectively alkylates all reduced thiols in PTEN, but not oxidized thiols or thiols modified by Michael addition with reactive electrophiles. Samples were washed in 1 ml of O_2_-free extraction buffer then transferred to a 15-ml conical tube. After adding SDS to a final concentration of 1% v/v, the mixture was held 2 h at 25°C in the dark, and proteins were precipitated with TCA [trichloroacetic acid], 10% v/v for 1 h. The precipitate was washed twice with acetone to remove traces of TCA, NEM, and IAA. Precipitated proteins were solubilized and oxidized or modified cys residues were reduced in 0.1 ml of O_2_-free reducing buffer (50 mM Hepes-NaOH, pH 7.7, 1 mM EDTA, 2% SDS and 4 mM DTT) for 30 min at 50°C. Reduced proteins were subsequently biotinylated with 0.9 ml of a solution containing 50 mM NaHPO_4_ pH 7.0, 1 mM EDTA, and 1 mM biotin conjugated to polyethylene oxide-maleimide for 30 min at 50°C. Proteins were precipitated in 10% v/v TCA for 1 h. The precipitate was isolated by centrifugation, washed with dry ice-chilled acetone, and solubilized in 0.3 ml of 50 mM Hepes-NaOH, pH 7.7, 1 mM EDTA, and 2% SDS. The sample was then diluted with 0.3 ml of the same solution without SDS. 15 µg protein was assayed by immunoblot for PTEN. A separate sample (200 µg protein) was added to 100 µL immobilized NA beads in 1 ml PBS, 0.4% v/v Tween 20. This suspension was rotated 16 hr at 4°, centrifuged, and beads were washed twice with PBS/0.4% v/v Tween 20. Loading buffer (50 µL) with 5% BME was added directly to beads, boiled for 10 min to release maleimido-biotinylated proteins, and 20 µL was assayed by immunoblot for total and oxidized PTEN.

### Identification of PTEN occurring as an intra-molecular disulfide

MCF-7 cells (MEM 1% v/v FBS) treated for 30 min with 20 µM Δ12-PGJ_2_, 4-HNE, acrolein, 15-HpETE or for 10 min with 100 µM H_2_O_2_ were lysed and proteins (15 µg) in the lysates were fractionated by non-reducing SDS-10% PAGE, followed by anti-PTEN immunoblot to distinguish native PTEN from oxidized PTEN occurring as an intra-molecular Cys^124^-Cys^71^ disulfide [28].

### Identification of PTEN covalently modified by cyclopentenone PG-biotin analogs

MCF-7 cells (MEM 1% v/v FBS) treated 1 hr with 1–10 µM of the aminopentylbiotinamide analogs of PGA_1_ or Δ12 PGJ_2_, were lysed, sonicated 10× for 1 s at 4°C, then centrifuged 10,000× g for 10 min. Supernatant with 100 µg of protein was incubated with 100 µl of NA beads in 1 ml PBS with 0.4% Tween 20 for 16 h at 4°C to sequester proteins containing a biotin epitope introduced *de novo* by reaction with cyPG-biotin analogs. The beads were then centrifuged at 500× g for 5 min to isolate neutravidin-biotin complexes (NA pulldown). The beads were washed 3× with 1 ml of PBS/0.4%Tween 20 then boiled 5 min in Laemmli loading buffer with 5% BME to release bound proteins. These samples were analyzed by immunoblotting for PTEN or proteins with a biotin epitope.

### Western immunoblotting

Following treatment with reactive electrophiles, H_2_O_2_, or enzyme inhibitors, MCF-7 or HEK-EBNA cells were lysed in 250 mM sucrose, 50 mM Tris pH 7.4, 5 mM MgCl_2_, 1 mM EGTA, 1× Complete™ protease inhibitor, 2 mM NaF and 2 mM sodium orthovanadate. Samples were dissolved in 50 µl of Laemmli loading buffer, 0.5% BME and heated at 95°C for 10 min. Samples (15–30 µg protein) were fractionated by SDS-PAGE and transferred to PVDF membranes. Membranes were blocked with 5% w/v nonfat dry milk in TBS-T, then incubated for 16 h at 4°C with primary antibodies directed against PTEN (1∶1000), biotin (1∶10,000), Akt (1∶1000), phospho-(T^308^)Akt (1∶1000), phospho-(S^473^)Akt (1∶1000), phospho-(S^241^)PDK1 (1∶1000), GSK3β (1∶1000), phospho-(S^9^)-GSK3β (1∶1000), RxRxx-phospho-(S/T) (1∶1000) or β-catenin (1∶1000) followed by HRP-conjugated secondary antibody (1∶5000). Antigen-antibody complexes were detected with Western Lightning™ ECL reagents. The intensity of chemiluminescent protein-antibody complexes was quantified with a Kodak Image Station 440™. Bar graphs depict the mean ± S.E. from densitometric analyses of separate experiments.

### Akt phosphorylation and signaling

∼1×10^6^ MCF-7 cells (MEM 1% v/v FBS) were treated with 0–20 µM Δ12-PGJ_2_ for 30 min at 37°C, or with 20 µM Δ12-PGJ_2_ for 0–120 min to determine concentration and time dependence. ∼1×10^6^ MCF-7 cells were also treated 30 min at 37°C with 10 µM of various PGs, including PGD_2_ and its cyPG dehydration products PGJ_2_, Δ12-PGJ_2_ and 15-deoxy-Δ12, Δ14-PGJ_2_; PGE_2_ and its cyPG dehydration products PGA_2_, its epimer 8-iso-PGA_2_, and its isomer PGB_2_ to determine structure-activity relationships. In certain experiments cells were also treated with 50 µM LY294002, 5 µM troglitazone, or 10 µM Akt inhibitor IV. Lysates from treated cells (15 µg protein) were fractionated on SDS-10% PAGE and proteins were transferred to PVDF membranes for immunoblot analysis of Akt, phospho-(T^308^)Akt, phospho-(S^473^)Akt, Akt substrate proteins containing the K/R-x-K/R-x-x-S/T(PO_4_) epitope, phospho-(S^241^)PDK1, and PTEN.

### Wnt/β-catenin signaling in STF3A cells

STF cells are HEK-293 cells containing a stably integrated SuperTopFlash (STF) transgene with TCF binding sites upstream of a luciferase reporter gene [59]. STF cells have negligible β-catenin/TCF-LEF transactivation and luciferase expression unless they are exposed to a Wnt ligand, e.g. WNT3A. We derived a subsidiary cell line, designated STF3A, by transfecting parental STF cells with a linearized pPGK + Wnt3A plasmid and a linearized blasticidin resistance plasmid for cell selection. STF cells grown on 10-cm plates were transfected using LipofectAMINE 2000 (Invitrogen). At 24 h after transfection, cells were serially diluted, re-plated and grown in medium with blasticidin (10 µg/ml) for 14 d. Forty colonies were screened for β-catenin/TCF-LEF activity by measuring luciferase activity normalized to total protein concentration. The selected STF3A clones, which stably expressed and secreted WNT3A, were maintained at 37°C in DMEM with 10% FBS, penicillin/streptomycin and 10 µg/ml blasticidin in a humidified incubator with 5% CO_2_. We measured wnt/β-catenin signaling in STF3A cells by quantifying their Super Topflash® luciferase reporter signal. STF3A cells (20,000/well) were plated in white, clear bottom 96-well plates coated with poly-L-lysine and grown 24 h at 37°C. Medium was removed and replaced with 200 µl fresh medium containing 0–300 ng/ml DKK1; 2–20 µM of α,β-enone containing cyPGs; 2–20 µM of α,β-enal metabolites derived from lipid peroxidation (acrolein, MDA, crotonaldehyde, 4-HNE, 4-ONE); 20 µM of primary PGs, PGH_2_, PGE_2_, PGF_2_α, PGD_2_, PGI_2_; 20 µM BSO, a glutathione synthesis inhibitor; other inhibitors, or DMSO vehicle. STF3A cells were incubated for 24 h at 37°C, washed with 200 µl PBS at 4°C, and lysed with 20 µl lysis buffer. Luciferin (60 µl/well) was added and luciferase activity in the lysates was quantified by fluorimetry. LDH activity in the lysate, an index of cell count, was quantified by spectroscopy. The ratio of luciferase/LDH activity is proportional to nuclear β-catenin signaling in STF3A cells.

### Inhibition of PTEN Enzymatic Activity

Inhibition of PTEN with 0–30 µM reactive electrophiles was quantified by using a PTEN enzyme assay kit (# 17–351, Upstate Biotechnology).

### Statistical analysis

Statistical significance at p<0.05 was assessed by analysis of variance (ANOVA) with Bonferroni's post-hoc test for comparisons among groups.

## References

[1] Peek RM, Mohla S, DuBois RN (2005). Inflammation in the genesis and perpetuation of cancer: summary and recommendations from a national cancer institute-sponsored meeting.. Cancer Res.

[2] Mantovani A, Allavena P, Sica A, Balkwill F (2008). Cancer-related inflammation.. Nature.

[3] Balkwill F, Charles KA, Mantovani A (2005). Smoldering and polarized inflammation in the initiation and promotion of malignant disease.. Cancer Cell.

[4] Medzhitov R (2008). Origin and physiological roles of inflammation.. Nature.

[5] Roncucci L, Mora E, Mariani F, Bursi S, Pezzi A (2008). Myeloperoxidase-positive cell infiltration in colorectal carcinogenesis as indicator of colorectal cancer risk.. Cancer Epidemiol Biomarkers Prev.

[6] Sheehan KM, Sheahan K, O'Donoghue DP, MacSweeney F, Conroy RM (1999). The relationship between cyclooxygenase-2 expression and colorectal cancer.. Jama.

[7] Rostom A, Dube C, Lewin G, Tsertsvadze A, Barrowman N (2007). Nonsteroidal anti-inflammatory drugs and cyclooxygenase-2 inhibitors for primary prevention of colorectal cancer: a systematic review prepared for the U.S. Preventive Services Task Force.. Ann Intern Med.

[8] Piazza GA, Alberts DS, Hixson LJ, Paranka NS, Li H (1997). Sulindac sulfone inhibits azoxymethane-induced colon carcinogenesis in rats without reducing prostaglandin levels.. Cancer Res.

[9] Carothers AM, Moran AE, Cho NL, Redston M, Bertagnolli MM (2006). Changes in antitumor response in C57BL/6J-Min/+ mice during long-term administration of a selective cyclooxygenase-2 inhibitor.. Cancer Res.

[10] Nathan C (2002). Points of control in inflammation.. Nature.

[11] Gilroy DW, Colville-Nash PR, McMaster S, Sawatzky DA, Willoughby DA (2003). Inducible cyclooxygenase-derived 15-deoxy(Delta)12-14PGJ2 brings about acute inflammatory resolution in rat pleurisy by inducing neutrophil and macrophage apoptosis.. Faseb J.

[12] Rajakariar R, Hilliard M, Lawrence T, Trivedi S, Colville-Nash P (2007). Hematopoietic prostaglandin D2 synthase controls the onset and resolution of acute inflammation through PGD2 and 15-deoxyDelta12 14 PGJ2.. Proc Natl Acad Sci U S A.

[13] Serhan CN, Chiang N, Van Dyke TE (2008). Resolving inflammation: dual anti-inflammatory and pro-resolution lipid mediators.. Nat Rev Immunol.

[14] Fitzpatrick FA, Wynalda MA (1983). Albumin-catalyzed metabolism of prostaglandin D2. Identification of products formed in vitro.. J Biol Chem.

[15] Buckley CD, Pilling D, Lord JM, Akbar AN, Scheel-Toellner D (2001). Fibroblasts regulate the switch from acute resolving to chronic persistent inflammation.. Trends Immunol.

[16] Shibata T, Kondo M, Osawa T, Shibata N, Kobayashi M (2002). 15-deoxy-delta 12,14-prostaglandin J2. A prostaglandin D2 metabolite generated during inflammatory processes.. J Biol Chem.

[17] Serhan CN, Savill J (2005). Resolution of inflammation: the beginning programs the end.. Nat Immunol.

[18] Esterbauer H, Schaur RJ, Zollner H (1991). Chemistry and biochemistry of 4-hydroxynonenal, malonaldehyde and related aldehydes.. Free Radic Biol Med.

[19] West JD, Marnett LJ (2006). Endogenous reactive intermediates as modulators of cell signaling and cell death.. Chem Res Toxicol.

[20] Grimsrud PA, Xie H, Griffin TJ, Bernlohr DA (2008). Oxidative stress and covalent modification of protein with bioactive aldehydes.. J Biol Chem.

[21] Anderson MM, Hazen SL, Hsu FF, Heinecke JW (1997). Human neutrophils employ the myeloperoxidase-hydrogen peroxide-chloride system to convert hydroxy-amino acids into glycolaldehyde, 2-hydroxypropanal, and acrolein. A mechanism for the generation of highly reactive alpha-hydroxy and alpha,beta-unsaturated aldehydes by phagocytes at sites of inflammation.. J Clin Invest.

[22] Uchida K, Kanematsu M, Morimitsu Y, Osawa T, Noguchi N (1998). Acrolein is a product of lipid peroxidation reaction. Formation of free acrolein and its conjugate with lysine residues in oxidized low density lipoproteins.. J Biol Chem.

[23] Esterbauer H, Eckl P, Ortner A (1990). Possible mutagens derived from lipids and lipid precursors.. Mutat Res.

[24] Chen Y, Morrow JD, Roberts LJ (1999). Formation of reactive cyclopentenone compounds in vivo as products of the isoprostane pathway.. J Biol Chem.

[25] Rossi A, Kapahi P, Natoli G, Takahashi T, Chen Y (2000). Anti-inflammatory cyclopentenone prostaglandins are direct inhibitors of IkappaB kinase.. Nature.

[26] Di Cristofano A, Pandolfi PP (2000). The multiple roles of PTEN in tumor suppression.. Cell.

[27] Clevers H (2006). Wnt/beta-catenin signaling in development and disease.. Cell.

[28] Lee SR, Yang KS, Kwon J, Lee C, Jeong W (2002). Reversible inactivation of the tumor suppressor PTEN by H2O2.. J Biol Chem.

[29] Leslie NR, Bennett D, Lindsay YE, Stewart H, Gray A (2003). Redox regulation of PI 3-kinase signalling via inactivation of PTEN.. Embo J.

[30] Moos PJ, Edes K, Cassidy P, Massuda E, Fitzpatrick FA (2003). Electrophilic prostaglandins and lipid aldehydes repress redox-sensitive transcription factors p53 and hypoxia-inducible factor by impairing the selenoprotein thioredoxin reductase.. J Biol Chem.

[31] Gayarre J, Stamatakis K, Renedo M, Perez-Sala D (2005). Differential selectivity of protein modification by the cyclopentenone prostaglandins PGA1 and 15-deoxy-Delta12,14-PGJ2: role of glutathione.. FEBS Lett.

[32] Landar A, Shiva S, Levonen AL, Oh JY, Zaragoza C (2006). Induction of the permeability transition and cytochrome c release by 15-deoxy-Delta12,14-prostaglandin J2 in mitochondria.. Biochem J.

[33] Downward J (1998). Mechanisms and consequences of activation of protein kinase B/Akt.. Curr Opin Cell Biol.

[34] Cantley LC, Neel BG (1999). New insights into tumor suppression: PTEN suppresses tumor formation by restraining the phosphoinositide 3-kinase/AKT pathway.. Proc Natl Acad Sci U S A.

[35] Plas DR, Thompson CB (2005). Akt-dependent transformation: there is more to growth than just surviving.. Oncogene.

[36] Maehama T, Dixon JE (1998). The tumor suppressor, PTEN/MMAC1, dephosphorylates the lipid second messenger, phosphatidylinositol 3,4,5-trisphosphate.. J Biol Chem.

[37] Shahabi NA, Chegini N, Wittliff JL (1987). Alterations of MCF-7 human breast cancer cell after prostaglandins PGA1 and PGF2 alpha treatment.. Exp Cell Biol.

[38] Chinery R, Coffey RJ, Graves-Deal R, Kirkland SC, Sanchez SC (1999). Prostaglandin J2 and 15-deoxy-delta12,14-prostaglandin J2 induce proliferation of cyclooxygenase-depleted colorectal cancer cells.. Cancer Res.

[39] Narumiya S, Fukushima M (1986). Site and mechanism of growth inhibition by prostaglandins. I. Active transport and intracellular accumulation of cyclopentenone prostaglandins, a reaction leading to growth inhibition.. J Pharmacol Exp Ther.

[40] van Noort M, Meeldijk J, van der Zee R, Destree O, Clevers H (2002). Wnt signaling controls the phosphorylation status of beta-catenin.. J Biol Chem.

[41] Coombs GS, Covey TM, Virshup DM (2008). Wnt signaling in development, disease and translational medicine.. Curr Drug Targets.

[42] Huang W, Chang HY, Fei T, Wu H, Chen YG (2007). GSK3 beta mediates suppression of cyclin D2 expression by tumor suppressor PTEN.. Oncogene.

[43] Persad S, Troussard AA, McPhee TR, Mulholland DJ, Dedhar S (2001). Tumor suppressor PTEN inhibits nuclear accumulation of beta-catenin and T cell/lymphoid enhancer factor 1-mediated transcriptional activation.. J Cell Biol.

[44] Hays E, Schmidt J, Chandar N (2009). Beta-catenin is not activated by downregulation of PTEN in osteoblasts.. In Vitro Cell Dev Biol Anim.

[45] Shin SY, Chin BR, Lee YH, Kim JH (2006). Involvement of glycogen synthase kinase-3beta in hydrogen peroxide-induced suppression of Tcf/Lef-dependent transcriptional activity.. Cell Signal.

[46] McCulloch MW, Coombs GS, Banerjee N, Bugni TS, Cannon KM (2009). Psammaplin A as a general activator of cell-based signaling assays via HDAC inhibition and studies on some bromotyrosine derivatives.. Bioorg Med Chem.

[47] Castellone MD, Teramoto H, Williams BO, Druey KM, Gutkind JS (2005). Prostaglandin E2 promotes colon cancer cell growth through a Gs-axin-beta-catenin signaling axis.. Science.

[48] Yin Y, Shen WH (2008). PTEN: a new guardian of the genome.. Oncogene.

[49] Gericke A, Munson M, Ross AH (2006). Regulation of the PTEN phosphatase.. Gene.

[50] Okumura K, Mendoza M, Bachoo RM, DePinho RA, Cavenee WK (2006). PCAF modulates PTEN activity.. J Biol Chem.

[51] Kwon J, Lee SR, Yang KS, Ahn Y, Kim YJ (2004). Reversible oxidation and inactivation of the tumor suppressor PTEN in cells stimulated with peptide growth factors.. Proc Natl Acad Sci U S A.

[52] Connor KM, Subbaram S, Regan KJ, Nelson KK, Mazurkiewicz JE (2005). Mitochondrial H2O2 regulates the angiogenic phenotype via PTEN oxidation.. J Biol Chem.

[53] Covey TM, Edes K, Fitzpatrick FA (2007). Akt activation by arachidonic acid metabolism occurs via oxidation and inactivation of PTEN tumor suppressor.. Oncogene.

[54] Finkel T, Holbrook NJ (2000). Oxidants, oxidative stress and the biology of ageing.. Nature.

[55] Wagner TM, Mullally JE, Fitzpatrick FA (2006). Reactive lipid species from cyclooxygenase-2 inactivate tumor suppressor LKB1/STK11: cyclopentenone prostaglandins and 4-hydroxy-2-nonenal covalently modify and inhibit the AMP-kinase kinase that modulates cellular energy homeostasis and protein translation.. J Biol Chem.

[56] Cernuda-Morollon E, Pineda-Molina E, Canada FJ, Perez-Sala D (2001). 15-Deoxy-Delta 12,14-prostaglandin J2 inhibition of NF-kappaB-DNA binding through covalent modification of the p50 subunit.. J Biol Chem.

[57] Zhang R, Brennan ML, Shen Z, MacPherson JC, Schmitt D (2002). Myeloperoxidase functions as a major enzymatic catalyst for initiation of lipid peroxidation at sites of inflammation.. J Biol Chem.

[58] Dvorak HF (1986). Tumors: wounds that do not heal. Similarities between tumor stroma generation and wound healing.. N Engl J Med.

[59] Xu Q, Wang Y, Dabdoub A, Smallwood PM, Williams J (2004). Vascular development in the retina and inner ear: control by Norrin and Frizzled-4, a high-affinity ligand-receptor pair.. Cell.

